# Integrating network pharmacology, transcriptomics, molecular docking and in vitro experiments to investigate the material basis and mechanism of Liuwei Dihuang Pills for treating diabetic nephropathy

**DOI:** 10.29219/fnr.v69.12763

**Published:** 2025-10-23

**Authors:** Chaoqun Liu, Xiaopeng Zhao, Anlei Yuan, Jiaye Tian, Bin Yu, Zewen Wang, Zhenzhen Xu, Yanxia Liu, Shijie Bi, Liansheng Qiao, Zhaozhou Lin, Yanling Zhang

**Affiliations:** a Key Laboratory of TCM-information Engineer of State Administration of TCM, School of Chinese Materia Medica, Beijing University of Chinese Medicine, Beijing 102488, China; b Beijing Tongrentang Co., Ltd., Beijing 100000, China

**Keywords:** Liuwei Dihuang Pills, *diabetic nephropathy*, *material basis*, *mechanism*

## Abstract

**Background:**

Diabetic nephropathy (DN) is a common microvascular complication of diabetes. *Liuwei Dihuang Pills* (LW) has significant clinical efficacy in the treatment of DN, but its pharmacological mechanism and material basis remain poorly understood.

**Objective:**

This study aimed to explore the substance basis and mechanism of action of LW in treating DN.

**Design:**

The potential mechanism of LW was investigated through UPLC/LTQ-Orbitrap-MS and network pharmacology. The activity of LW and its constituent herbs was evaluated in human renal glomerular endothelial cells (HRGEC) and HK2 cells. Key pathways and targets were identified by transcriptomics and validated by qRT-PCR. Molecular docking was employed to screen for active ingredients of LW.

**Results:**

The results showed that LW, Cornus Officinalis (CO), Moutan Cortex (MC), Rhizoma Dioscoreae (RD), and Alismatis Rhizoma (AR) mitigated oxidative damage in HRGEC cells. LW primarily targeted VEGFA and EGR1, thereby modulating the AGE-RAGE signaling pathway in diabetic complications. LW also reduced fibrosis in HK2 cells by up-regulating BMP4 and modulating the TGF-beta signaling pathway. Rehmanniae Radix Praeparata (RRP), CO, MC, RD, and Poria Cocos (PC) were identified as key contributors to improving renal fibrosis. Additionally, 43 potential active ingredients were identified in LW, 13 of which exhibited favorable ADMET properties. Six key ingredients, including taxifolin, cianidanol, gallic acid, caffeic acid, 5-hydroxymethylfurfural, and paeonol were found to be primarily responsible for the effects of LW on microvascular endothelial injury and renal fibrosis.

**Conclusion:**

In summary, these findings reveal the material basis and mechanism of LW against DN, providing a foundation for its clinical application.

## Popular scientific summary

- Unveiling How Liuwei Dihuang Pills Battle Diabetic Kidney Damage
- Diabetic nephropathy (DN) is a common and serious complication of diabetes. While Liuwei Dihuang Pills (LW), a traditional Chinese medicine, have shown promise in treating it, how exactly they work has remained a mystery.
- In this study, researchers set out to uncover the active ingredients in LW and understand how they combat DN. Using advanced techniques like mass spectrometry and network pharmacology, they explored the pills’ potential mechanisms.
- The study found that LW and its constituent herbs, including Cornus Officinalis (CO), Moutan Cortex (MC), Rhizoma Dioscoreae (RD), and Alismatis Rhizoma (AR), can effectively alleviate oxidative damage in renal vascular cells. This protective effect on the renal microvasculature is primarily achieved by modulating two key targets: VEGFA and EGR1.
- Furthermore, the study addressed renal fibrosis, a critical step in kidney disease progression. It demonstrated that LW can ameliorate fibrosis by upregulating the BMP4 protein and inhibiting the TGF-β signaling pathway. Key contributors to this anti-fibrotic effect include Rehmanniae Radix Praeparata (RRP), CO, MC, RD, and Poria Cocos (PC).
- The researchers also pinpointed 43 potential active ingredients in LW, with 13 showing good properties for drug development. Among these, six ingredients stood out as the main contributors to LW’s effects on kidney health.
- In short, this study sheds light on how LW can protect the kidneys in diabetes, paving the way for their wider clinical use.

Diabetic nephropathy (DN), a prevalent microvascular complication of diabetes, manifests as pathological kidney changes and stands as a leading cause of renal failure (1). It is reported that 20%~40% diabetic patients suffer from DN, which poses significant threats to their physical and mental health (2). Current pharmacological interventions for DN, such as hypoglycemic, antihypertensive, lipid-lowering, and antioxidant agents (3), have made progress in treatment. However, they are often limited by side effects and incomplete efficacy, highlighting the urgent need for alternative therapeutic strategies.

*Liuwei Dihuang Pills* (LW), a well-known traditional Chinese medicine (TCM) prescription composed of *Rehmanniae Radix Praeparata* (RRP), *Cornus Officinalis* (CO), *Moutan Cortex* (MC), *Rhizoma Dioscoreae* (RD), *Alismatis Rhizoma* (AR) and *Poria Cocos* (PC) has been widely used in DN treatment (4). Clinical studies have shown that LW is safe and effective in treating DN proteinuria (5). Studies have reported that LW can protect mesangial cells and prevent renal fibrosis in DN rats (6). Shi et al. (7) also have found that LW has a practical therapeutic effect on DN. Despite these findings, the precise mechanism of LW in DN treatment remains unclear due to its multi-compound and multi-target nature, representing a significant research gap in the field.

**Fig. 1:**
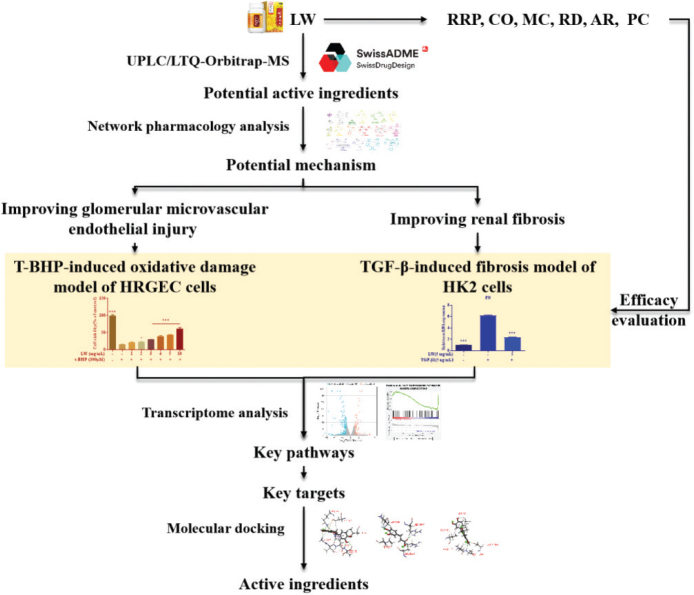
Work scheme of integrating network pharmacology, transcriptomics, molecular docking and *in vitro* experiments to investigate the material basis and mechanism of *Liuwei Dihuang Pills* for treating diabetic nephropathy.

To address this gap, this study aimed to systematically elucidate the potential mechanism and material basis of LW against DN. We employed a multi-method approach. Firstly, *in vitro* gastrointestinal simulated digestion, a well-established technique in food and drug research that mimics the human digestive process without the need for animal ethical approval (8), was used to simulate the digestion of LW in the gastrointestinal tract. Then, UPLC/LTQ-Orbitrap-MS and network pharmacology (9, 10) were employed to elucidate the potential mechanism of LW against DN. Secondly, the potential mechanism was verified by *in vitro* experiments, and its key pathways and targets were analyzed by transcriptome analysis (11, 12). Finally, molecular docking was applied in this study to discover the material basis of LW for DN treatment (13). In this study, the material basis of LW treatment of DN were discovered by molecular docking (Fig. 1).

## Material and methods

### Reagents

LW, RRP, CO, MC, RD, AR and PC were bought from Tong Ren Tang Technologies Co., Ltd. (Beijing, China). Tert-butyl Hydroperoxide (t-BHP), KCl, KH_2_PO_4,_ NaHCO_3_, NaCl, MgCl_2_·6H_2_O, (NH_4_)_2_CO_3_, CaCl_2_ were obtained from Macklin (Shanghai, China). Pepsin was bought from Sigma-Aldrich (St. Louis., MO, USA). Bile salts were obtained from Beijing Solarbio Science & Technology Co., Ltd. (Beijing, China). Isopropanol, chloroform, and absolute ethanol were purchased from Beijing Chemical Industry Group CO., Ltd. (Beijing, China). Fetal bovine serum (FBS) and penicillin-streptomycin (PS) were provided by Gibco BRL (Grand Island, NY, USA). Dulbecco’s modified eagle medium (DMEM) was purchased from Corning Incorporated (Corning, USA). Cell Counting Kit-8 (CCK-8) was acquired from Sigma-Aldrich (Madrid, Spain). TRIzol reagent was obtained by Invitrogen (Carlsbad, CA, USA).

### Preparation of LW, RRP, CO, MC, RD, AR and PC

LW, RRP, CO, MC, RD, AR and PC were ground into powder. Gastrointestinal simulated digestion was performed according to Minekus et al. (8). The composition of Simulated Gastric Fluid (SGF) and Simulated Intestinal Fluid (SIF) is shown in Table S1. They were diluted to a 1.25× concentration with ultrapure water. 10 g of LW, RRP, CO, MC, RD, AR, or PC powder was mixed with 75 mL of SGF, 16 mL of 12,500 U/mL pepsin, and 50 μL of 0.15 M CaCl_2_. Then, ultrapure water were added to the mixture until the total volume reached 100 mL. The mixture was adjusted to pH = 3 with 1 M HCl and incubated with shaking at 37 °C (200 rpm/min) for 2 h. After digestion, the pH was adjusted to 7 to obtain the gastric digest. The gastric digest was mixed with 55 mL SIF, 25 mL 800 U/mL trypsin, 114 mg bile salts, 200 μL 0.3 M CaCl_2_, and ultrapure water was added to 100 mL. The mixture was adjusted to pH 7 with 1 M NaOH and incubated with shaking at 37 °C (200 rpm/min) for 2 h. After the reaction was complete, the final mixture was heated in a 98 °C water bath for 10 min to halt the pancreatic enzyme reaction. Subsequently, the digestion product was centrifuged at 10,000 rpm for 10 min. The supernatants were dried and stored at −20°C .

### Characterization of ingredients in LW by UPLC/LTQ-Orbitrap-MS

The test solution was prepared by dissolving the gastrointestinal simulated digestion product of LW in 50% methanol to a concentration of 100 mg/mL and filtered through a 0.22 μm membrane. Sample analysis was performed on an Accela 600 high-performance liquid chromatography system coupled with a LTQ-Orbitrap mass spectrometry (UPLC/LTQ-Orbitrap-MS) system. The chromatographic and mass spectrometric conditions are shown in Table S2. The sample of LW was analyzed using LTQ Orbitrap XL mass spectrometer and the total ion chromatograms of the corresponding compounds were extracted by Xcalibur 2.1 workstation. Identities of the compounds were obtained by matching their retention times (RT), molecular ions and product ions with literature data.

### Analysis of the potential mechanism of LW by network pharmacology

#### Prediction of LW targets

The structures of ingredients in LW were obtained from PubChem (https://pubchem.ncbi.nlm.nih.gov/) in SDF format. SwissADME (http://www.swissadme.ch/) (14) was used to predict the druggability of ingredients. Chemical ingredients that exhibited ‘High’ GI absorption and satisfied at least three of Lipinski’s five rules were selected as the potential active ingredients in LW. All predicted targets of potential active ingredients in LW were harvested using the SwissTargetPrediction (http://www.swisstargetprediction.ch/) (15) and Discovery Studio 4.0. The species in the SwissTargetPrediction was set to ‘Homo sapiens’ to obtain the predicted target. While for Discovery Studio 4.0, the pharmacophore model of human targets in PharmaDB was used to find the targets of ingredients.

#### Collection of DN-related targets

The DN-related targets were collected from GeneCards database (https://www.genecards.org/) (16) and DisGeNET database (https://www.disgenet.org/) (17). Targets with a score greater than average in GeneCards database and DisGeNET database were selected as DN-related targets.

#### PPI network construction

To identify the potential targets of LW in the treatment of DN, we screened the intersection targets of LW-related and DN-related targets by Venny 2.1.0 (https://bioinfogp.cnb.csic.es/tools/venny/index.html) (18). The STRING database (https://cn.string-db.org/) (19) was used to collect the protein–protein interactions (PPI) data for these potential targets. And we set confidence score >0.4 to ensure the reliability of the PPI data included in our analysis. Then, the PPI data from the STRING database were imported into Cytoscape 3.7.1 software, and thereby its visualization function was used to construct a PPI network for LW treatment of DN. We adjusted the layout settings to clearly display the relationships between nodes (representing potential targets) and edges (representing PPI).

#### GO enrichment analysis

To discover the biological functions involved in LW treatment of DN, GO enrichment analysis of PPI network was performed using the ClueGO plugin (20) in Cytoscape3.7.1, with the species limited to ‘Homo sapiens’ and *P* < 0.05.

### The potential mechanism was verified by in vitro experiments

#### Cell culture and treatment

Human renal glomerular endothelial cells (HRGEC) were obtained from Shang Hai Ze Ye Biotechnology Co., Ltd. (Shanghai, China). Human renal tubule epithelial cells (HK2) were provided by Wuhan Pricella Biotechnology Co., Ltd. (Wuhan, China). Cells were incubated in a humidified incubator at 5% CO_2_ and 37°C. HRGEC cells were cultured in DMEM containing 10% FBS and 1% PS. HK2 cells were cultured in MEM plus 10% FBS and 1% PS.

#### CCK-8 assay

HRGEC cells (8 × 10^3^ cells/well) were seeded in 96-well plates. HRGEC cell injury was induced by 300 μM t-BHP. The gastrointestinal digestion products of LW, RRP, CO, MC, RD, AR, and PC were dissolved in PBS. They were then prepared at a concentration of 100 mg/mL. Cell viability was detected by CCK – 8 assay. This was to evaluate the protective effect of LW (1–10 mg/mL), RRP (1.25–5 mg/mL), CO (0.313–1.25 mg/mL), MC (1.25–5 mg/mL), RD (2.5–10 mg/mL), AR (2.5–10 mg/mL), and PC (1.25–5 mg/mL) on oxidative stress injury of HRGEC. After treatment, 100 μL CCK-8 was added to each well, then the absorbance at 450 nm was measured by flex station 3 (Molecular Devices, San Francisco, CA, USA) after 2 h.

#### Quantitative real-time PCR

HRGEC cells and HK2 cells were seeded in 6 – well plates at a density of 5 × 10^5^ and 2 × 10^5^ cells/well, respectively. The cells were divided into control, model and drug treatment groups. For HK2 cells, the model group was treated with 5 ng/mL Transforming Growth Factor (TGF)-β1 for 72 h, and the drug groups were given different concentrations of LW, RRP, CO, MC, RD, AR and PC. Subsequently, RNA of the cells was isolated using TRIzol reagent. Reverse transcription was conducted according to the instruction of the PrimeScript RT kit. SYBR Premix Ex Taq reagent was used to detect mRNA expression, and the 2-ΔΔCt method was applied to perform mRNA fold changes. The primers sequences of objective genes were presented in Table S3.

### Discovery of key pathways and targets by transcriptome analysis

Total RNA was extracted using TRIzol reagent and RNA quantification and quality control were performed using the 5400 BioAnalyzer (Agilent, USA). Library preparation and sequencing for transcriptome analysis were completed by Novogene Technology Co., Ltd. (Beijing, China). Principal component analysis (PCA) was performed on the control group, the model group, and the drug group based on gene expression levels. Differential expression analysis between each group was performed using the DESeq2 R package. Genes with |log2FC| > 1 and *P*-value< 0.05 were identified as differentially expressed genes (DEGs). All genes affected by LW were subjected to pathway enrichment analysis using GSEA (https://www.gsea-msigdb.org/gsea/index.jsp) to explore the enriched pathways of LW-affected genes. Using KEGG gene sets as the annotation background, pathways with *P* < 0.05 and False Discovery Rate (FDR) < 0.25 were considered significantly enriched. KEGG enrichment analysis of LW callback-DEGs was performed using DAVID database (https://david.ncifcrf.gov/) (21). The common pathways identified through both Gene Set Enrichment Analysis (GSEA) and Kyoto Encyclopedia of Genes and Genomes (KEGG) analyses were selected as key pathways regulated by LW. Callback-DEGs in core enrichment list of key pathways were obtained as key targets.

### Explore the material basis through molecular docking

To explore the material basis of LW in the treatment of DN, we evaluated the interaction between the potential active ingredients in LW (Section ‘Prediction of LW targets’) and key targets by molecular docking. The structure of the potential active ingredients was optimized using the Prepare Ligands and Minimize Ligands parts of Discover Studio 4.0, including hydrogen atom treatment, charge distribution and energy minimization. The 3D structure of the targets was selected in the RCSB PDB database (https://www.rcsb.org/) or AlphaFold (https://alphafold.ebi.ac.uk/). The Discover Studio 4.0 was used to construct molecular docking model. Docking poses were clustered using a RMSD threshold of 2.0 Å. The detailed docking parameter are summarized in Table S5. And the ingredients were docked with key targets by CDOCKER.

### Statistical analysis

The experimental data were expressed as mean ± standard deviation (SD). The results were analyzed by one-way analysis of variance (ANOVA). All experiments in this study were repeated at least three times independently. *P* < 0.05 was considered statistically significant.

## Results

### Chemical profiling of gastrointestinal simulated digestion product of LW using UPLC/LTQ-Orbitrap-MS

**Fig. 2:**
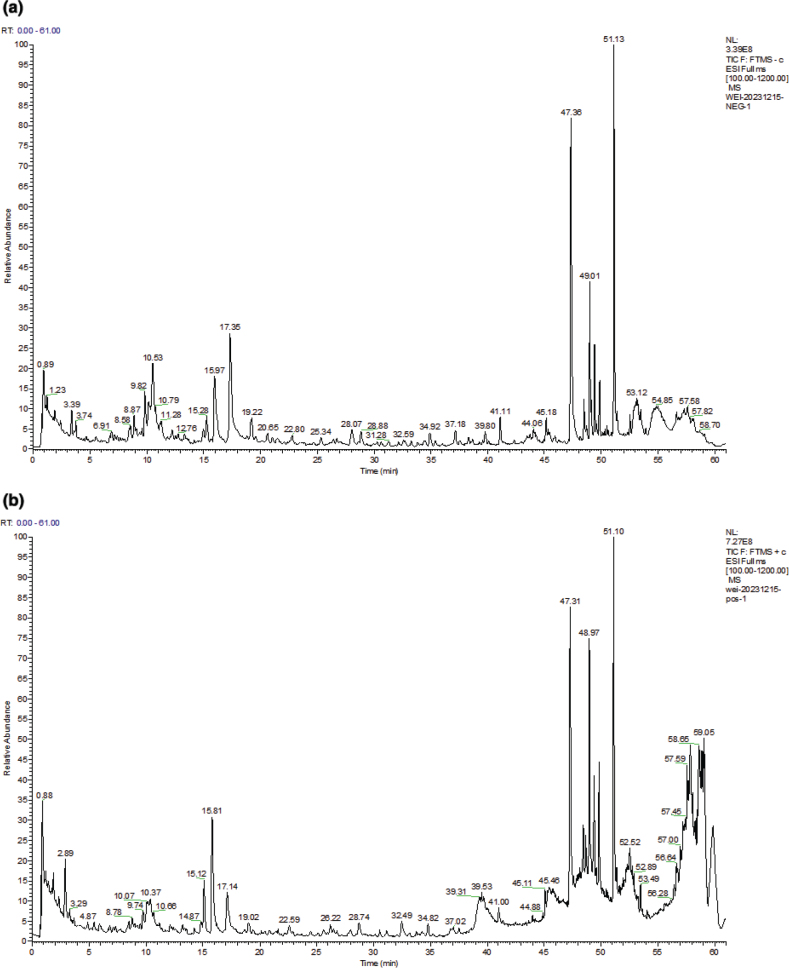
Total ion chromatograms of gastrointestinal simulated digestion product of LW in negative ion mode (a) and positive ion mode (b).

In this study, UPLC/LTQ-Orbitrap-MS was employed to identify the chemical ingredients in the gastrointestinal simulated digestion product of LW. The total ion chromatograms in positive and negative ion modes are shown in Fig. 2. A total of 43 prototype components were identified (Table 1), including 10 iridoid glycosides, 4 triterpenoids, 6 monoterpenoids and 4 phenylethanoid glycosides.

**Table 1:** Identification of gastrointestinal simulated digestion product of LW

No.	RT (min)	Component	Formula	Ion	Error (ppm)	Calculated mass (m/z)	Fragmentation Information
1	0.98	Quinic acid	C_7_H_12_O_6_	[M-H]-	4.373	191.05585	126.88950,110.861600,110.861600
2	1.17	Citric Acid	C_6_H_8_O_7_	[M-H]-	2.256	191.01906	190.82870, 172.93642, 154.85179, 92.76472, 84.76959, 66.85500
3	1.27	Caffeic Acid	C_9_H_8_O_4_	[M+H]+	1.296	181.04977	163.03998, 145.05029
4	1.43	Adenosine	C_10_H1_3_N_5_O_4_	[M+H]+	−0.24	268.10379	136.06189, 119.03571
5	1.74	Isocitric acid	C_6_H_8_O_7_	[M-H]-	2.989	191.0192	190.95662, 110.88416, 86.86577, 84.71971, 58.80437
6	1.8	Gallic Acid	C_7_H_6_O_5_	[M-H]-	4.735	169.01395	124.86752, 96.88680
7	2.92	5-Hydroxymethylfurfural	C_6_H_6_O_3_	[M+H]+	−2.681	127.03863	126.69484, 108.75797
8	3.7	Rehmannioside D	C_27_H_42_O_20_	[M-H]-	−0.189	685.21844	113.0237
9	4.36	Mannitol	C_6_H_14_O_6_	[M-H]-	4.614	181.0715	162.96730, 100.93450, 88.74340, 70.87910, 58.85310
10	4.67	Gardoside	C_16_H_22_O_10_	[M-H]-	2.269	373.1138	210.92900, 166.97470, 148.93980, 122.85870
11	6.24	Aucubin	C_15_H_22_O_9_	[M-H]-	0.815	345.1183	345.07907, 299.15012, 179.05582, 151.03938, 119.03381, 89.02303, 73.02802, 59.01251
12	7.27	Loganin	C_17_H_26_O_10_	[M-H]-	2.278	389.1449	343.08670, 180.91480, 112.99720
13	8.41	Cianidanol	C_15_H_14_O_6_	[M-H]-	0.285	289.07095	245.08311, 125.02327, 245.08311
14	8.47	Loganic acid	C_16_H_24_O_10_	[M-H]-	1.377	375.1291	213.00710, 168.86470, 150.93200, 142.88950, 106.95290
15	9.82	8-Epiloganic acid	C_16_H_24_O_10_	[M-H]-	0.898	375.12891	212.94900, 168.97220, 151.00190, 112.90020
16	10.04	Genipin	C_11_H_14_O_5_	[M+H]+	−1.277	227.09111	208.98958
17	10.53	Oxypaeoniflora	C_23_H_28_O_12_	[M-H]-	1.812	495.1506	465.14550, 333.14260, 165.02090
18	14.77	Catalpin	C_22_H_26_O_12_	[M+H]+	3.727	483.14557	405.22318, 465.03195
19	15.12	Sweroside	C_16_H_22_O_9_	[M+H]+	−2.029	359.13293	358.72540, 196.97141, 150.97229, 126.85617
20	15.28	Acetylcatalpol	C_17_H_24_O_11_	[M-H]-	1.32	403.1239	357.18440, 194.97680, 178.92830, 124.89660
21	15.91	Ethyl benzoylformate	C_10_H_10_O_3_	[M+H]+	−0.283	179.07022	178.96851, 160.94868, 132.99019, 104.93394
22	17.35	Mudanpioside E	C_24_H_30_O_13_	[M-H]-	2.119	525.161	479.10300, 449.07570, 164.99770
23	19.22	Perseitol heptacetate isometric	C_21_H_30_O_14_	[M-H]-	1.465	505.1561	227.0305
24	20.72	Echinacoside	C_35_H_46_O_20_	[M-H]-	1.29	785.25116	623.22278
25	21.49	Ellagic acid	C_14_H_6_O_8_	[M-H]-	2.247	300.9986	301.08030, 256.94270, 201.02390, 184.91280
26	22.54	1,2,3,6-Tetragalloylglucose	C_34_H_28_O_22_	[M-H]-	−0.811	787.0983	617.23180, 483.1008, 6450.17370, 423.10960
27	24.38	Taxifolin	C_15_H_12_O_7_	[M-H]-	0.993	303.05023	178.99153
28	24.7	6’-O-Galloyl paeoniflorin	C_30_H_32_O_15_	[M-H]-	0.322	631.1659	399.16520, 375.12890, 313.16640, 270.96390, 241.07640, 211.00550
29	26.34	Azelaic Acid	C_9_H_16_O_4_	[M-H]-	4.033	187.0974	187.10520, 168.91730, 124.95230
30	27.08	Verbascoside	C_29_H_36_O_15_	[M-H]-	−0.235	623.1969	461.16705, 161.02359, 135.04471
31	27.99	Mudanpioside H	C_30_H_32_O_14_	[M-H]-	0.501	615.171	220.98750, 190.97840, 136.97490
32	28.15	Pentagalloylglucose	C_41_H_32_O_26_	[M-H]-	−0.657	939.1093	617.16200, 601.20040, 447.10030
33	28.84	Jioglutoside B1/B2 isometric	C_37_H_50_O_20_	[M-H]-	0.025	813.2812	619.36150, 491.33030, 473.27060
34	28.91	Isoacteoside	C_29_H_36_O_15_	[M-H]-	−0.235	623.1969	461.16663, 179.03403
35	34.92	Mudanpioside C	C_30_H_32_O_13_	[M-H]-	0.989	599.1766	447.14120, 281.07750, 148.85140, 136.85710
36	36.19	Paeonol	C_9_H_10_O_3_	[M+H]+	−1.201	167.07007	167.01091, 148.91283, 120.97849
37	37.15	Benzoyloxypeoniflorin	C_30_H_32_O_13_	[M-H]-	1.206	599.1766	429.2271
38	41.11	Mudanpioside J	C_31_H_34_O_14_	[M-H]-	1.332	629.1873	431.1061
39	45	16-Oxoalisol A	C_30_H_48_O_6_	[M+H]+	−2.129	505.35129	487.33218, 469.36087, 451.27170, 415.35471, 397.41617
40	48.34	Alisol A	C_30_H_50_O_5_	[M-H]-	1.428	489.3578	489.17218, 471.22015
41	48.58	11-deoxyalisol isometric	C_30_H_46_O_4_	[M+H]+	−1.159	471.34634	453.42410, 435.33536, 381.33829, 339.26764
42	50.48	Linolelaidic acid	C_18_H_30_O_2_	[M+H]+	−0.06	279.23184	279.11826, 261.15826, 233.17484, 219.24664, 205.06683, 177.05630, 163.03427
43	55.74	Ursolic Acid	C_30_H_48_O_3_	[M-H]-	0.106	455.35202	411.32888

### The potential mechanism of LW in the treatment of DN

**Fig. 3:**
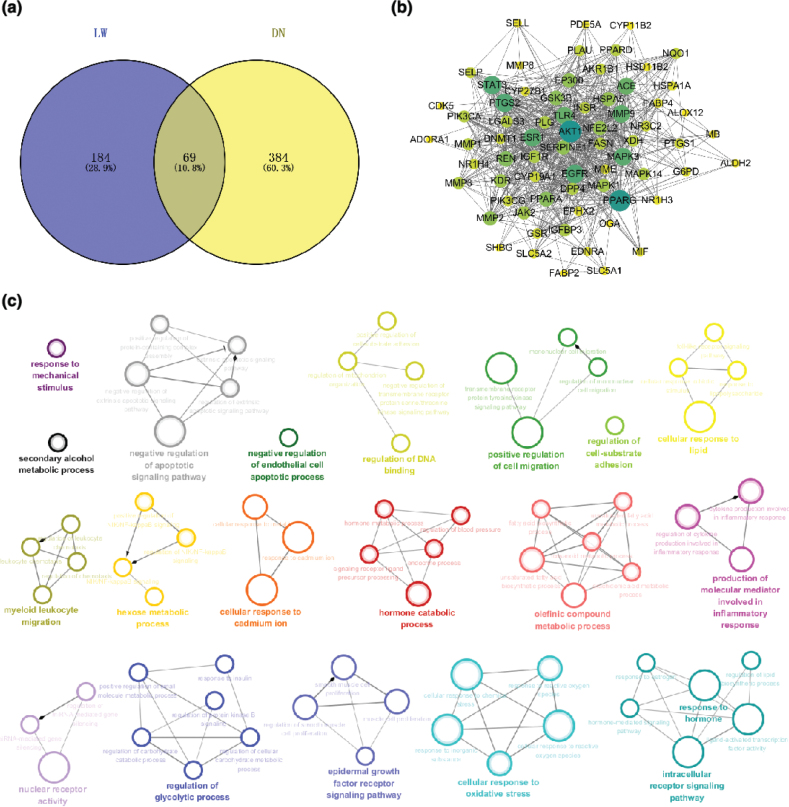
Target determination and network pharmacology analysis of LW in the treatment of DN. (a) Venn diagram of overlapped targets between LW potential targets and DN targets. (b) PPI of potential targets of LW to treat DN. Nodes are potential targets for LW in the treatment of DN. The larger the node, the deeper the color, the greater the Degree. The line between two nodes represents the interaction. (c) Results of GO enrichment analysis.

A total of 13 potential active ingredients in LW were identified using SwissADME (Table S4). A total of 253 LW-related targets were predicted using SwissTargetPredition and Discovery Studio 4.0, while 453 DN-related targets were obtained from GeneCards and DisGeNET. Based on the Venny2.1.0, 69 overlapping targets were recognized as potential targets for LW treatment of DN (Fig. 3a). Subsequently, PPI network for LW treatment of DN (Fig. 3b) were constructed using the STRING database and Cytoscape3.7.1, which contains 69 nodes and 645 edges. GO enrichment analysis of the PPI network, performed using the ClueGO plug, identified 270 GO-BP entries. Cluster analysis of these GO-BP entries resulted in 19 functional sets (Fig. 3c). The results showed that the main biological processes involved in the LW treatment of DN were negative regulation of apoptotic signaling pathway, epidermal growth factor receptor signaling pathway, regulation of cell-substrate adhesion, cellular response to oxidative stress, myeloid leukocyte migration and production of molecular mediator involved in inflammatory response. These biological processes were associated with inflammation, oxidative stress, cell proliferation-death regulation and fibrosis. Studies have revealed that glomerular microvascular endothelial cell damage caused by oxidative stress and inflammation can lead to glomerular capillary dysfunction, an early marker of DN (22). Hyperglycemia can cause renal tubular epithelial cells to release various cytokines, such as epidermal growth factor and transforming growth factor, which can cause renal tubular epithelial-mesenchymal transition and eventually lead to renal fibrosis (the main pathological manifestation of DN) (23). Therefore, inhibition of glomerular microvascular endothelial injury and renal fibrosis may be the key mechanisms through which LW exerts its therapeutic effects in DN.

### In vitro efficacy evaluation of LW

The results of network pharmacology analysis showed that LW could resist glomerular microvascular endothelial injury and renal fibrosis by affecting various biological processes, thereby treating DN. Therefore, in this study, t-BHP was used to establish a microvascular endothelial oxidative damage model in HRGEC cells (24), while TGF-β was used to establish a fibrosis model in HK2 cells (25). Then the efficacy of LW was evaluated by these two models.

**Fig. 4:**
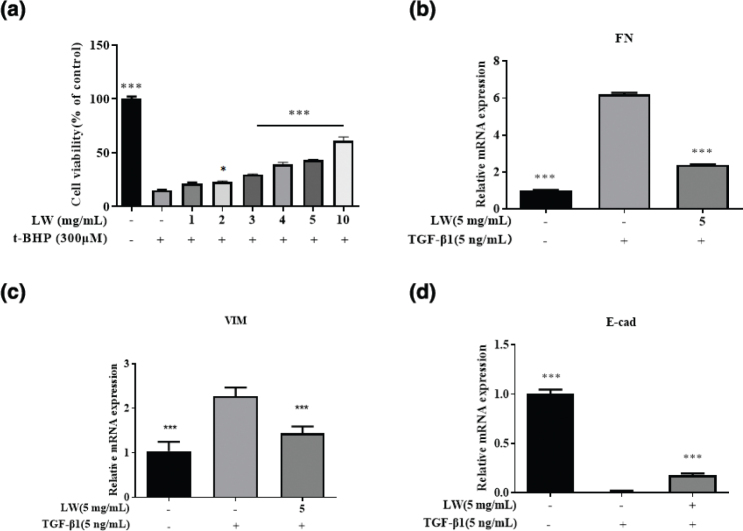
*In vitro* efficacy verification results of LW. (a) The protective effect of LW on t-BHP-induced HRGEC cells. (b–d) FN, VIM, and E-cad mRNA levels in HK2 cells. Data are expressed as mean ± SD (*n* = 3, *P < 0.05, ***P < 0.001 vs. model group).

The protective effect of LW on renal glomerular endothelial injury was assessed, as shown in Fig. 4a. We found that LW (1–10 mg/mL) could effectively inhibit the oxidative stress injury of HRGEC cells induced by t-BHP. The effect of LW on renal tubular fibrosis was evaluated by TGF-β1-induced HK2 cells fibrosis model. As shown in Fig. 4b–d, compared with the model group, LW could increase the mRNA expression of *E-cad*, and decrease the mRNA expression of *FN* and *VIM*, indicating that LW could significantly improve the fibrosis of HK2 cells.

### In vitro efficacy evaluation of RRP, CO, MC, RD, AR and PC

**Fig. 5:**
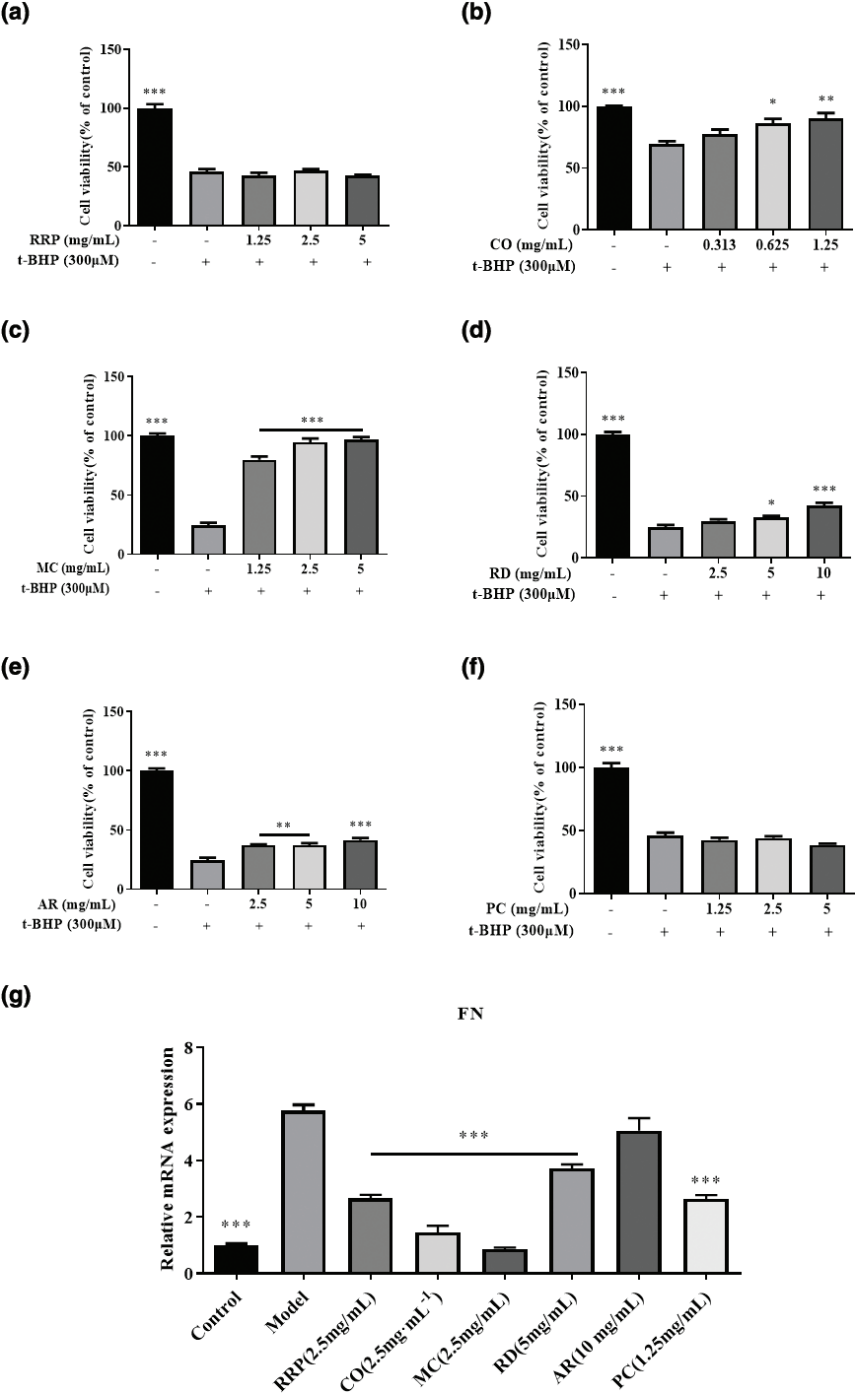
*In vitro* efficacy verification results of RRP, CO, MC, RD, AR and PC. (a–f) The protective effect of RRP, CO, MC, RD, AR and PC on t-BHP-induced HRGEC cells. (g) FN mRNA levels in HK2 cells. Data are expressed as mean ± SD (*n* = 3, *P < 0.05, **P < 0.01, ***P < 0.001 vs. model group).

This study further evaluated the efficacy of RRP, CO, MC, RD, AR and PC, which are the constituent herbs of LW. We found that CO (0.625–1.25 mg/mL), MC (1.25–5 mg/mL), RD (5–10 mg/mL) and AR (2.5–10 mg/mL) significantly protected the microvascular oxidative stress injury of HRGEC (Fig. 5a–f). However, RRP and PC had no protective effect on microvascular endothelial injury. The experimental results showed that except for AR, 2.5 mg/mL RRP, 2.5 mg/mL CO, 2.5 mg/mL MC, 5 mg/mL RD and 1.25 mg/mL PC could significantly improve the fibrosis of HK2 cells (Fig. 5g).

### Transcriptome analysis results of LW improving HRGEC cells injury

**Fig. 6:**
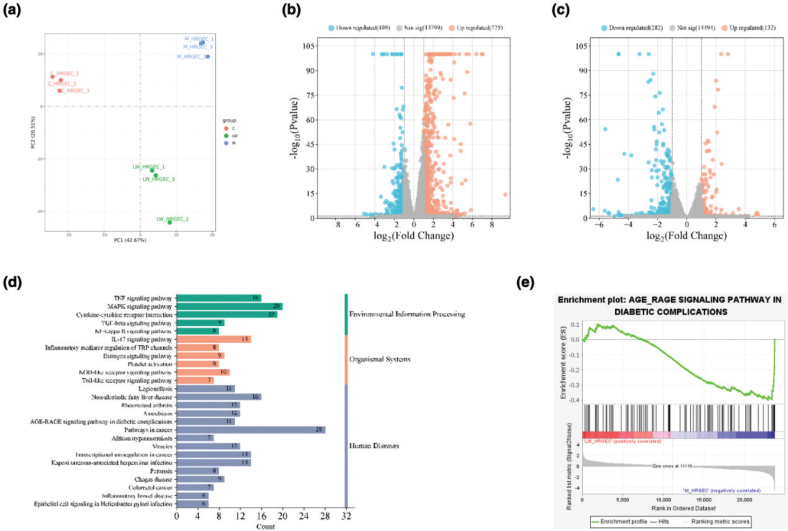
Transcriptome analysis. (a) The results of PCA analysis. (b) The volcano map showed DEGs in model versus control groups. (c) The volcano map showed DEGs in LW versus model groups. (d) Common pathways obtained by GSEA and KEGG analysis. (e) AGE-RAGE signaling pathway in diabetic complications.

In order to analyze the mechanism of LW improving microvascular endothelial injury, we performed transcriptome analysis based on the oxidative stress injury model of HRGEC cells. PCA analysis showed (Fig. 6a) three main clusters including control group, model group and LW (5 mg/mL) group. A total of 1,184 DEGs were screened by comparing model and control groups (Fig. 6b), including 775 upregulated genes and 409 downregulated genes. A total of 414 DEGs were screened by comparing LW and model groups (Fig. 6c), including 132 upregulated genes and 282 downregulated genes. Among the DEGs regulated by LW, 192 were callback-DEGs.

GSEA was used to perform functional enrichment of all genes in LW-treated HRGEC cells to investigate its effects on oxidative damage, identifying 99 significantly enriched pathways (*P* < 0.05 and FDR < 0.25). A total of 49 pathways were significantly enriched (*P* < 0.05) by KEGG enrichment analysis of LW callback-DEGs. A total of 26 common pathways were obtained by GSEA and KEGG analysis (Fig. 6d), including AGE-RAGE signaling pathway in diabetic complications, Tumor Necrosis Factor (TNF) signaling pathway, IL-17 signaling pathway, MAPK signaling pathway and Cytokine-cytokine receptor interaction, etc. Among them, AGE-RAGE signaling pathway in diabetic complications (Fig. 6e) was closely related to microvascular oxidative damage. Studies have found that the binding of AGE to its cell surface receptor (RAGE) can trigger microvascular damage and accelerate the pathogenesis of DN (26, 27). Vascular Endothelial Growth Factor A (VEGFA) and Early Growth Response 1 (EGR1) were identified as key targets for LW to regulate AGE-RAGE signaling pathway in diabetic complications, which were core callback-DEGs in this pathway (Fig. S1A). The results suggest that LW may downregulate the AGE-RAGE signaling pathway in diabetic complications to treat DN by regulating VEGFA and EGR1.

### Transcriptome analysis results of LW inhibiting HK2 cells fibrosis

**Fig. 7:**
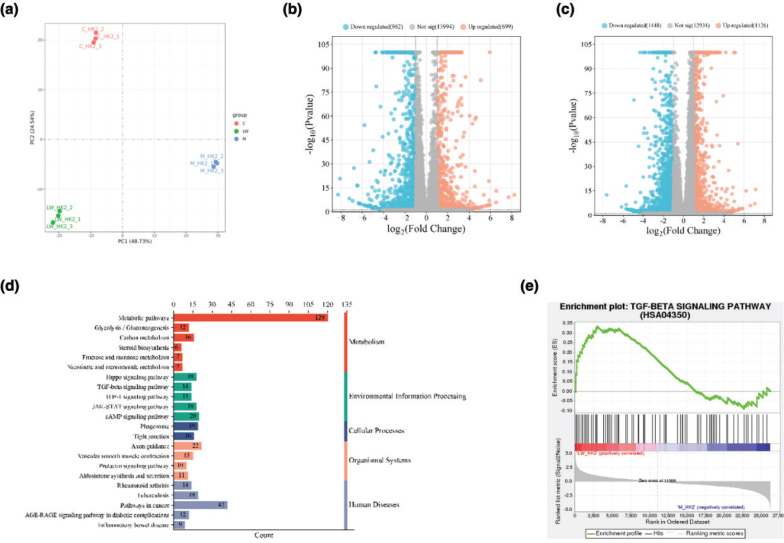
Transcriptome analysis. (a) The results of PCA analysis. (b) The volcano map showed DEGs in model versus control groups. (c) The volcano map showed DEGs in LW versus model groups. (d) Common pathways obtained by GSEA and KEGG analysis. (e) TGF-beta signaling pathway.

To investigate the mechanism of LW improving renal fibrosis, we performed transcriptome analysis based on HK2 cell fibrosis model. PCA revealed (Fig. 7a) three main clusters including control group, model group and LW (2 mg/mL) group. A total of 1,661 DEGs were screened by comparing model and control groups (Fig. 7b), including 699 upregulated genes and 962 downregulated genes. A total of 2,574 DEGs were screened by comparing LW and model groups (Fig. 7c), including 1,126 upregulated genes and 1,448 downregulated genes. Among the DEGs regulated by LW, 996 were callback-DEGs.

All genes of LW ameliorating fibrosis in HK2 cells were used for GSEA analysis, and 210 pathways were obtained (*P* < 0.05 and FDR < 0.25). A total of 30 pathways were significantly enriched (*P* < 0.05) by KEGG enrichment analysis of LW callback-DEGs. 22 common pathways were obtained by GSEA and KEGG analysis (Fig. 7d), such as TGF-beta signaling pathway, HIF-1 signaling pathway, JAK-STAT signaling pathway and Glycolysis/Gluconeogenesis. Among them, TGF-beta signaling pathway (Fig. 7e) was closely related to renal fibrosis. Studies have shown that TGF-β is a key mediator of renal fibrosis and induces renal fibrosis mainly by activating its downstream signaling pathways (28). BMP4 was a key callback-DEGs for LW to regulate the TGF-beta signaling pathway (Fig. S1B), suggesting that LW may upregulate the TGF-beta signaling pathway to treat DN by regulating BMP4.

### Key gene verification

**Fig. 8:**
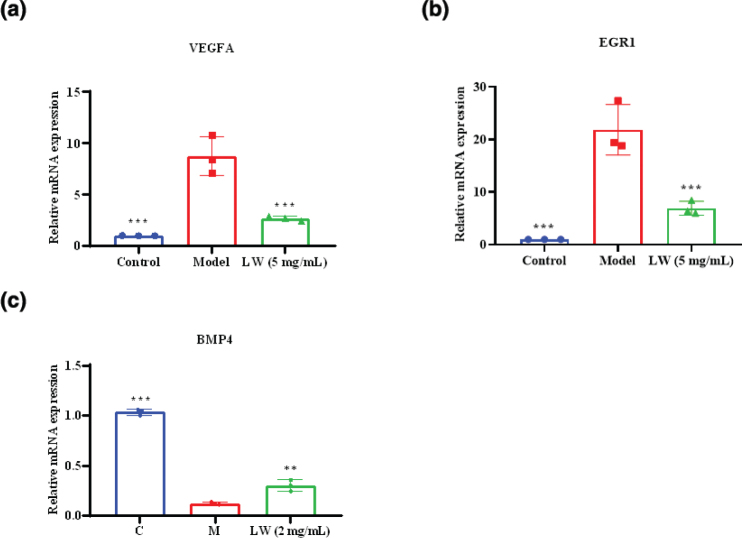
Results of key target validation (a) The mRNA expression of VEGFA. (b) The mRNA expression of EGR1. (c) The mRNA expression of BMP4. Data are expressed as mean ± SD (*n* = 3, **P < 0.01, ***P < 0.001 vs. model group).

We used Reverse Transcription Quantitative Polymerase Chain Reaction (RT-qPCR) to evaluate the regulatory effects of LW on the key targets VEGFA, EGR1, and BMP4 in HRGEC cells and HK2 cells. The results showed that LW significantly downregulated the mRNA expression of *VEGFA* and *EGR1* in HRGEC cells (Fig. 8a, b). These results indicated that LW could modulate the AGE-RAGE signaling pathway by downregulating the expression of *VEGFA* and *EGR1*. In HK2 cells, LW significantly upregulated the mRNA expression of *BMP4* (Fig. 8c), indicating that LW regulates TGF-beta signaling pathway by upregulating *BMP4*. In summary, these experimental findings are consistent with the transcriptome analysis results.

### Key ingredients discovery

The potential active ingredients of LW acting on key targets were screened by molecular docking. VEGFA (PDB ID: 1BJ1) and EGR1 (PDB ID: 4X9J) were used to screen active ingredients in CO, MC, RD and AR for their potential to improve microvascular injury. Additionally, BMP4 (AlphaFold) was selected to screen the active ingredients of RRP, CO, MC, RD and PC, which have the potential to improve renal fibrosis. The compounds with both -CDCOKER ENERGY > 0 and -CDCOKER INTERACTION ENERGY > 0 were selected as the key compounds.

**Fig. 9:**
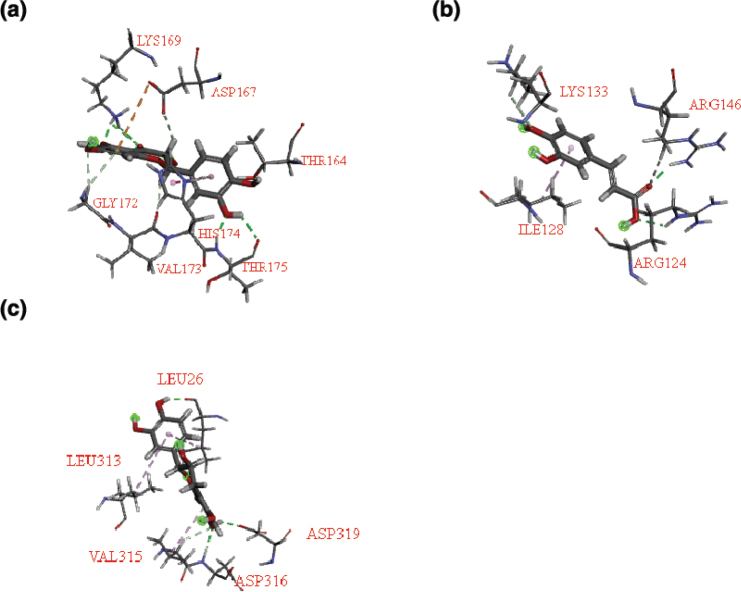
Molecular docking pattern diagrams. (a) Taxifolin interacting with VEGFA. (b) Caffeic acid interacting with EGR1. (c) Cianidanol interacting with BMP4.

The results (Table 2) showed that five ingredients had a good bonding activity with VEGFA, including taxifolin (Fig. 9a). Five ingredients, including caffeic acid (Fig. 9b), had a good binding affinity with EGR1. These results suggested that taxifolin and caffeic acid, etc. are the key ingredients of LW in improving oxidative stress damage in HRGEC cells. Six ingredients showed good BMP4 binding, including cianidanol (Fig. 9c) and gallic acid, suggesting that they are key ingredients of LW in improving fibrosis of HK2 cells.

**Table 2:** The result of CDOCKER analysis

Target	Compound	-CDCOKER ENERGY (kcal/mol)	-CDCOKER INTERACTION ENERGY (kcal/mol)	Source
VEGFA	Taxifolin	31.1636	37.4416	RD
	Cianidanol	30.2051	40.6242	MC
	Gallic Acid	29.0491	27.6263	CO, MC
	Caffeic Acid	25.4442	28.8874	CO
	5-Hydroxymethylfurfural	21.7569	21.2586	AR
	Paeonol	17.1715	23.3603	MC
EGR1	Caffeic Acid	23.6078	24.4558	CO
	Taxifolin	22.5774	27.1108	RD
	Gallic Acid	22.4338	18.4209	CO, MC
	5-Hydroxymethylfurfural	21.8402	21.8239	AR
	Cianidanol	21.0262	27.4786	MC
	Paeonol	15.6135	21.5625	MC
BMP4	Cianidanol	20.7948	27.7179	MC
	Gallic Acid	17.9675	14.8016	RRP, CO, MC
	Taxifolin	17.6087	23.0601	RD
	Caffeic Acid	16.0617	16.2105	RRP, CO
	5-Hydroxymethylfurfural	11.2385	10.5844	RRP
	Paeonol	6.91364	12.2557	MC

## Discussion

DN, a prevalent microvascular complication in diabetic patients, stands as a major contributor to renal failure. Despite extensive research, current therapeutic options for DN remain limited, highlighting the need to explore alternative treatment strategies such as TCM. LW has been widely used in TCM clinical practice for many years. Pharmacological studies have confirmed that LW has a significant clinical effect on DN and can improve the renal function of patients (4). However, due to the multi-component nature of TCM prescriptions, fully elucidating the mechanisms of LW in the treatment of DN remains a challenging task. In this study, we employed an integrative approach combining network pharmacology, transcriptomics, molecular docking, and in vitro validation to systematically elucidate the mechanisms and active components of LW in ameliorating DN. Our findings have analyzed the mechanism and material basis of LW in improving the two DN iconic pathological features of glomerular microvascular endothelial injury and renal fibrosis, and provided new insights into its multi-target pharmacological effects.

### Mechanism of LW in improving glomerular microvascular endothelial injury and renal fibrosis

Network pharmacology analysis revealed that LW likely exerts its therapeutic effects through modulation of oxidative stress, inflammatory responses, and fibrotic pathways. These findings align with evidence that DN progression is driven by intertwined pathological mechanisms, including glomerular microvascular endothelial injury induced by oxidative stress or inflammation and aberrant activation of pro-fibrotic signaling cascades (29–31). This suggests the efficacy and mechanism of LW can be verified from the perspectives of improving glomerular microvascular endothelial injury and renal fibrosis.

Specifically, the downregulation of *VEGFA* and *EGR1* in HRGEC cells observed in our study provides mechanistic support for ameliorated glomerular microvascular endothelial damage effects of LW. While VEGFA is essential for physiological angiogenesis, its pathological overexpression in diabetic conditions exacerbates endothelial dysfunction and glomerular hyperfiltration (32, 33). Similarly, EGR1 has emerged as a critical mediator of diabetic microvascular complications through its regulation of inflammatory and fibrotic gene networks (34). Our demonstration that LW suppresses these key mediators extends previous clinical observations (4) by identifying specific molecular pathways involved.

The anti-fibrotic effects of LW in HK2 cells were associated with upregulation of *BMP4*, a known antagonist of TGF-β signaling (35, 36). This finding is particularly significant given the central role of TGF-β in promoting extracellular matrix accumulation in DN. The BMP/TGF-β axis imbalance has been increasingly recognized as a therapeutic target in renal fibrosis and our results position LW as a natural modulator of this critical pathway.

### Identification of active ingredients and their roles

Through molecular docking with key targets, we identified six key ingredients in LW, namely taxifolin, cianidanol, gallic acid, caffeic acid, 5-hydroxymethylfurfural, and paeonol, which act on VEGFA, EGR1, or BMP4. These ingredients are likely the primary active components of LW in alleviating DN by improving glomerular microvascular endothelial injury and renal fibrosis. These findings bridge the gap between TCM’s holistic approach and modern molecular pharmacology.

Taxifolin, the dominant ingredient of RD (37), has been reported to mitigate kidney pathology in diabetes rats (38). Paeonol and cianidanol were the main ingredients of MC (39), delay renal injury through antioxidant effects and have shown efficacy in mitigating diabetic renal fibrosis (40, 41). 5-hydroxymethylfurfural, the primary ingredient of RRP (42), has antioxidant, antiproliferative, cytoprotective and anti-inflammatory activities (43). Gallic acid and caffeic acid are derived from several herbs in LW, such as RRP and CO. It is reported that gallic acid improves DN by regulating oxidative stress, endoplasmic reticulum stress and fibrosis (44). Caffeic acid also possesses diverse physiological properties, including antioxidant, anti-inflammatory, immune-stimulating and anti-proliferative activities (45).

Interestingly, our study failed to identify active components from PC and AR, possibly due to limitations in ADMET prediction algorithms or the presence of uncharacterized bioactive metabolites. This underscores the complexity of TCM formulations and the need for more comprehensive analytical approaches.

### Study’s limitations and future directions

While our integrated approach provides valuable mechanistic insights into LW’s anti-DN effects, several limitations should be acknowledged. The reliance on *in vitro* models, though informative, cannot fully replicate the complexity of human DN pathophysiology. In the following study, a variety of *in vitro* and *in vivo* experiments should be carried out on the basis of the results of this study to further elucidate the mechanisms of LW against DN. While molecular docking identified six bioactive components, the absence of active compounds from PC and AR may reflect limitations in ADMET prediction algorithms or the presence of uncharacterized metabolites, highlighting the importance of employing more advanced analytical techniques, such as metabolomics or activity-guided fractionation, to uncover potential hidden actives. Furthermore, this study focused on individual targets and pathways but did not evaluate potential synergies among LW components, which could be addressed through combinatorial pharmacology studies.

## Conclusions

**Fig. 10:**
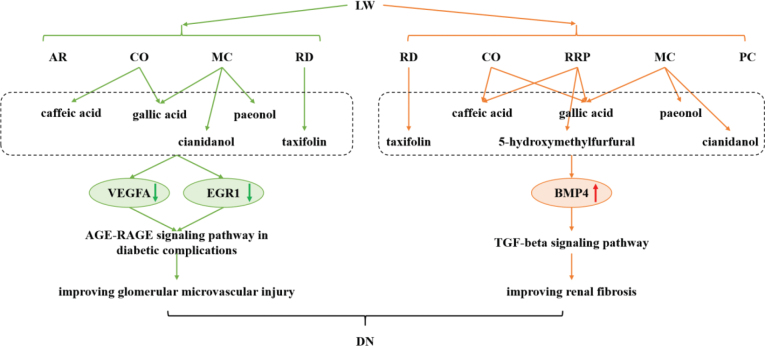
Schematic diagram of the potential mechanisms by which LW exerts its anti-DN effect.

In summary, this study provides evidence for understanding the mechanism and material basis of LW against DN. The results showed that LW regulated the AGE-RAGE signaling pathway in diabetic complications and the TGF-beta signaling pathway by targeting VEGFA, EGR1 and BMP4 through ingredients taxifolin, cianidanol, gallic acid, caffeic acid, 5-hydroxymethylfurfural and paeonol, thereby ameliorating glomerular microvascular injury and renal fibrosis (Fig. 10). These findings lay a foundation for the clinical application of LW in DN treatment.

## CRediT authorship contribution statement

Chaoqun Liu: Data curation, Visualization, Writing – original draft. Xiaopeng Zhao: Data curation. Anlei Yuan: Conceptualization, Data curation, Methodology. Jiaye Tian: Validation. Bin Yu: Validation. Zewen Wang: Formal analysis. Zhenzhen Xu: Investigation. Yanxia Liu: Investigation. Shijie Bi: Software. Liansheng Qiao: Visualization. Zhaozhou Lin: Supervision. Yanling Zhang: Writing – Review & Editing.

## Conflict of interest and funding

The authors declare no potential conflicts of interest.

## Data availability

Data will be made available on request.
